# Synaptic Potentiation in Hippocampus by eEF2K Inhibitor A484954

**DOI:** 10.1002/hipo.70091

**Published:** 2026-03-27

**Authors:** Qian Yang, Tian Li, Hannah M. Jester, Qiang Su, Xueyan Zhou, Alexey G. Ryazanov, Tao Ma

**Affiliations:** ^1^ Department of Internal Medicine, Gerontology and Geriatric Medicine Wake Forest School of Medicine Winston‐Salem North Carolina USA; ^2^ Department of Pharmacology Rutgers Robert Wood Johnson Medical School Piscataway New Jersey USA; ^3^ Department of Translational Neuroscience Wake Forest School of Medicine Winston‐Salem North Carolina USA

**Keywords:** eEF2K, eEF2K inhibitors, hippocampus, LTP, protein synthesis, synapses

## Abstract

An important mechanism controlling protein synthesis is through phosphorylation of the eukaryotic elongation factor 2 (eEF2) by its kinase eEF2K. Hyperphosphorylation of eEF2 is linked to many neuronal diseases characterized by cognitive impairments. Consistently, recent studies show that the inhibition of the eEF2K signaling via genetic or pharmacological approaches can alleviate synaptic failure and dementia syndromes in mouse models of Alzheimer's disease (AD) and related dementias (ADRDs). One commonly used tool to study eEF2K signaling is A‐484954 (or AG), a small molecule compound that is considered a highly selective and potent eEF2K antagonist. Here we reported that the AG compound (at three doses) can induce chemical long‐term potentiation (LTP) in acute hippocampal slices from mice. Taking advantage of two transgenic mouse models with eEF2K knockout or overexpression, we further demonstrated that eEF2K‐independent mechanisms contribute to chemical LTP induced by AG (dose‐dependent). Our data suggest cautious interpretation of findings on neuronal effects of eEF2K inhibitors such as AG. Future investigations are warranted to elucidate the detailed molecular mechanisms underlying the effects of AG compound and other eEF2K inhibitors on synaptic and cognitive function.

**Abbreviations**
ACSF: artificial cerebrospinal fluid
AD: Alzheimer's disease
AG: eEF2K Inhibitor A‐484954
DAB: 3,3‐diaminobenzidine
DMSO: dimethylsulfoxide
eEF2: eukaryotic elongation factor 2
eEF2K: eukaryotic elongation factor 2 Kinase
eIF2*α*
: eukaryotic initiation factor 2 alpha subunit
ELISA: enzyme‐linked immunosorbent assay
fEPSP: field excitatory post‐synaptic potential
GAPDH: glyceraldehyde‐3‐phosphate dehydrogenase
GTP: Guanosine triphosphate
LTP: long term potentiation
mRNA: messenger ribonucleic acid
mTOR: mammalian target of rapamycin
NH125: 1‐benzyl‐3‐cetyl‐2‐methylimidazolium iodide
PBS: phosphate‐buffered saline
PCR: polymerase chain reaction
PRP: plasticity‐related protein
WT: wildtype mice

## 1 Introduction

Protein synthesis (mRNA translation) entails three phases: initiation, elongation, and termination. Each phase involves complex machinery and is tightly regulated by specific translational factors (Hershey et al. 2019; Klann and Dever 2004; Sossin and Costa‐Mattioli 2019). The elongation phase, which consumes most (> 95%) of the energy and amino acids used in protein synthesis, is mainly controlled through phosphorylation of the eukaryotic elongation factor 2 (eEF2). Phosphorylation of eEF2 on the Thr56 site by its kinase eEF2K interferes with eEF2 engagement with ribosomes and results in inhibition of general mRNA translation (Liu and Proud 2016; Ma 2023; Ryazanov et al. 1988; Taha et al. 2013). Unlike the conventional protein kinases (CPKs) such as serine/threonine kinases and tyrosine kinases, eEF2K belongs to a small group of kinases termed “alpha‐kinases” or “*α*‐kinases,” whose catalytic domains are different from those in the CPKs (Drennan and Ryazanov 2004; Kenney et al. 2014; Ryazanov et al. 1999, 1997). Overactive eEF2K signaling is linked to many common diseases including cancer, cardiovascular diseases, and neuronal disorders (Liu and Proud 2016). Accumulating evidence indicates that regulation of eEF2K phosphorylation could be a key molecular mechanism underlying the antidepressant effects of ketamine (Adaikkan et al. 2018; Autry et al. 2011; Suzuki et al. 2021). Furthermore, a number of small molecule compounds have been developed as eEF2K inhibitors including A‐484954 (referred as AG hereafter), a selective and potent eEF2K antagonist that was originally developed by Abbott laboratories (Chen et al. 2011; Liu and Proud 2016).

Accumulating evidence over the past decade indicate a link between hyper‐phosphorylation of eEF2 and multiple neuronal diseases characterized by cognitive impairment such as Alzheimer's disease (AD), the most common dementia syndrome in the elderly (Beckelman et al. 2019; Jan et al. 2017; Kasica, Zhou, Jester, et al. 2022; Ma 2023; Ma et al. 2014). Recently we reported that systemic treatment with AG (through embedding AG pellets subcutaneously) can alleviate synaptic failure and cognitive deficits in aged AD and Down syndrome (DS) model mice (Kasica, Zhou, Yang, et al. 2022; Wang et al. 2024). We conducted electrophysiology experiments in ex vivo acute hippocampal slices to investigate the effects AG on long‐term potentiation (LTP), a major form of synaptic plasticity and cellular model for learning and memory (Bliss and Collingridge 1993; Malenka 2003). Surprisingly, we observed consistent potentiation of baseline field excitatory postsynaptic potentials (EPSPs) upon application of AG in slices from wild type (WT) mice in the absence of electrical stimulation that is, high‐frequency stimulation (HFS). We then conducted a series of synaptic electrophysiology experiments combined with pharmacogenetic approaches to understand this unusual chemical LTP and its underlying mechanisms. Particularly, we ask whether the AG‐induced chemical LTP is dependent on eEF2K by using two unique transgenic mouse models with eEF2K knockout or overexpression.

## 2 Results and Discussion

**FIGURE 1:**
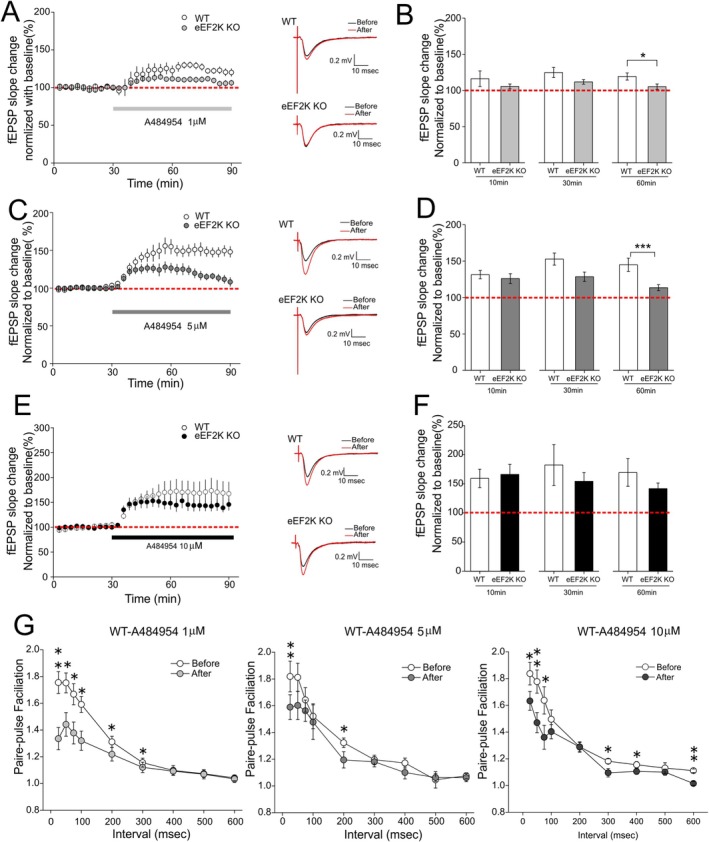
Chemical long‐term potentiation (LTP) induced by A484954 (AG) in hippocampal slices from both wild type (WT) and eEF2K knockout (eEF2K KO) mice. (A) Treatment of hippocampal slices with 1 μM AG induced modest LTP in WT (white, *n* = 8 slices from 4 mice) and eEF2K KO (gray, *n* = 8 slices from 4 mice) mice. Representative fEPSP traces before and after AG treatment (60 min after AG application) were shown. (B) Cumulative data showing mean fEPSP slopes 10, 30, and 60 min after AG application based on experimental results shown in A. (C) Treatment of hippocampal slices with 5 μM AG induced robust and sustained LTP in WT (white, *n* = 11 slices from 6 mice) mice but declined LTP in slices from eEF2K KO (dark gray, *n* = 9 slices from 4 mice) mice. Representative fEPSP traces before and after AG treatment (60 min after AG application) were shown. (D) Cumulative data showing mean fEPSP slopes 10, 30, and 60 min after AG application based on experimental results shown in C. (E) Treatment of hippocampal slices with 10 μM AG induced robust and sustained LTP in both WT (white, *n* = 6 slices from 3 mice) and eEF2K KO (black, *n* = 7 slices from 4 mice) mice. Representative fEPSP traces before and after AG treatment (60 min after AG application) were shown. (F) Cumulative data showing mean fEPSP slopes 10, 30, and 60 min after AG application based on experimental results shown in E. (G) Performance of paired‐pulse facilitation (PPF) was measured in WT slices treated with AG at 1 μM (*n* = 9 slices from 4 mice), 5 μM (*n* = 7 slices from 5 mice), and 10 μM (*n* = 7 slices from 3 mice). 2‐tailed independent Student's *t‐*test. *p < 0.05, **p < 0.01, ***p < 0.005.

After a baseline recording of field EPSPs for 30 min, we treated the hippocampal slices with AG at three concentrations: 1, 5, and 10 μM. In WT mice, the inhibitor at 1 μM immediately induced a mild yet consistent potentiation, average 125% and 119% of the baseline measured at 30 and 60 min after inhibitor application (Figure 1A,B). In slices derived from the eEF2K knockout mice (Beckelman et al. 2019), the potentiation induced by1 μM AG was blunted (average 112% and 105% of the baseline measure at 30 and 60 min after inhibitor application), though not completely to the baseline level until 60 min after the inhibitor application (*p* < 0.05, Figure 1A,B). In contrast, 5 μM AG induced significant and persistent LTP, average 153% and 148% of the baseline measured at 30 and 60 min post inhibitor treatment (Figure 1C,D). We next applied 5 μM AG on slices from the eEF2K KO mice and observed reduced LTP (average 126% and 128% of baseline measured at 10 min and 30 min post inhibitor treatment, respectively). Compared to WT slices, AG‐induced LTP was not significantly blunted until 60 min after compound treatment (average 113%, *p* < 0.01, Figure 1C,D). Finally, 10 μM AG treatment induced significant chemical LTP in slices from both WT and eEF2K KO mice (average 169% and 141% of baseline measured at 60 min post inhibitor treatment, respectively) (Figure 1E,F). As compared to the results with lower dose inhibitor (1 and 5 μM), chemical LTP induced by 10 μM AG in slices from the eEF2K KO mice was indistinguishable from that in WT mice. Additionally, we analyzed paired pulse facilitation (PPF), a calcium‐dependent short form of presynaptic plasticity (Santschi and Stanton 2003). We observed that overall PPF was reduced in slices treated with AG (at all three doses) compared to vehicle controls, particularly in PPF with shorter intervals (e.g., < 300 ms) (Figure 1G). We also assessed PPF performance in slices from the eEF2K KO mice treated with AG, and did not observe significant effects before and after drug treatment (Figure S1A,C,E). Taken together, these findings indicate that the chemical LTP induced by the eEF2K inhibitor AG is dose‐dependent, and could be attributed to, particularly for higher doses (> 5 μM), mechanisms that are independent of eEF2K inhibition.

**FIGURE 2:**
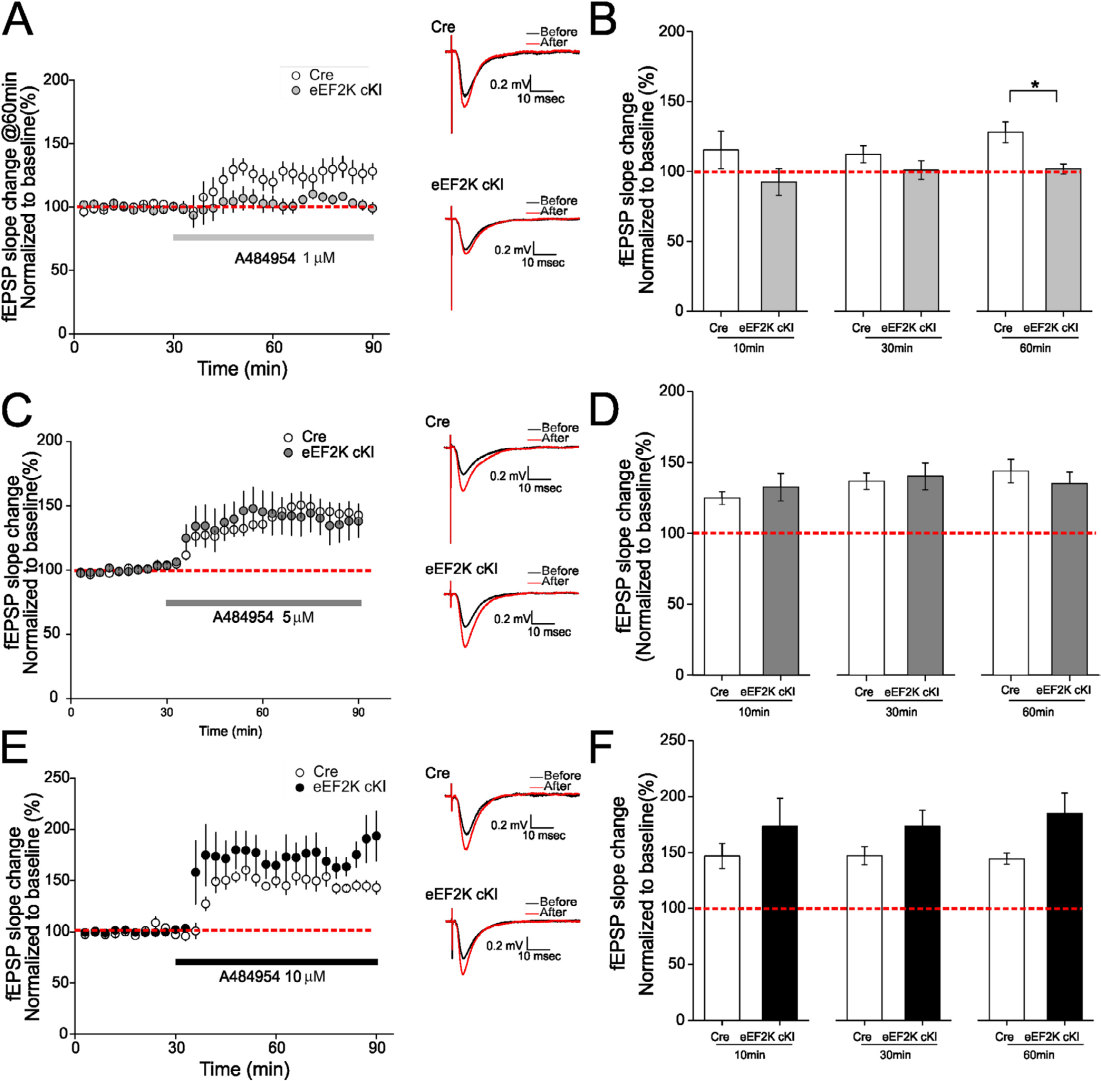
AG induced LTP in hippocampal slices from transgenic mice with eEF2K overexpression (eEF2K cKI). (A) Treatment with 1 μM AG failed to induce LTP in hippocampal slices from eEF2K cKI mice (gray, *n* = 6 slices from 3 mice). Modest LTP was induced in control (Cre) slices by 1 μM AG (white, *n* = 6 slices from 3 mice). Representative fEPSP traces before and after AG treatment (60 min after AG application) were shown. (B) Cumulative data showing mean fEPSP slopes 10, 30, and 60 min after AG application based on experimental results shown in A. (C) Treatment of hippocampal slices with 5 μM AG induced robust and sustained LTP in both Cre control (white, *n* = 7 slices from 6 mice) and eEF2K cKI (dark gray, *n* = 7 slices from 3 mice) mice. Representative fEPSP traces before and after AG treatment (60 min after AG application) were shown. (D) Cumulative data showing mean fEPSP slopes 10, 30, and 60 min after AG application based on experimental results shown in C. (E) Treatment of hippocampal slices with 10 μM AG induced robust and sustained LTP in both Cre control (white, *n* = 5 slices from 3 mice) and eEF2K cKI (black, *n* = 7 slices from 4 mice) mice. Representative fEPSP traces before and after AG treatment (60 min after AG application) were shown. (F) Cumulative data showing mean fEPSP slopes 10, 30, and 60 min after AG application based on experimental results shown in C. 2‐tailed independent Student's *t‐*test. *p < 0.05.

To further investigate the potential eEF2K‐independent mechanisms associated with AG‐induced chemical LTP, we took advantage of a novel transgenic mouse model with neuronal eEF2K overexpression (eEF2K cKI) developed in our lab. Briefly, we first created a line of transgenic mice with *Eef2K* knock‐in flanked by a *lox*STOP*lox* cassette using the CRISPR/Cas9‐mediated genome editing combined with a *Rosa26* knock‐in strategy (Chu et al. 2016)(in collaboration with Dr. Dale Cowley of the Animal Models Core Facility at the University of North Carolina at Chapel Hill). We then crossed these mice with the transgenic mice expressing a neuron‐specific Cre recombinase (Camk2a‐cre) (Yang et al. 2021) to remove the STOP cassette and thus induce conditional overexpression of eEF2K in excitatory neurons in the forebrain and hippocampus. The hypothesis to be tested is whether the hippocampal chemical LTP induced by AG, an established potent eEF2K inhibitor, can be reversed by upregulating neuronal eEF2K activities. In slices from the eEF2K cKI mice, 1 μM AG failed to induce chemical LTP as compared to the results from control Cre mice (Figure 2A). In contrast, we observed similar chemical LTP induced by AG compound at higher doses (5 and 10 μM) in slices from both Cre and eEF2K cKI mice (Figure 2C–F). Interestingly, at 60 min post inhibitor treatment, there was a trendy increase of chemical LTP in the eEF2K cKI slices compared to the control slices (*p* = 0.06). Additionally, we measured PPF in slices from the eEF2K cKI mice treated with AG and did not observe significant effects before and after drug treatment (Figure S1B,D,F). We also confirmed that treatment of AG decreased eEF2 phosphorylation in hippocampal slices from the eEF2K cKI mice (Figure S2). Such findings are in line with the results from the eEF2K KO mice (Figure 1), indicating an eEF2K‐independent component of chemical LTP induced by AG (dose‐dependent).

Overactivation of the eEF2K signaling has been linked to a number of common human diseases including cardiovascular diseases, cancer (solid tumors), and neurodegenerative diseases (Liu and Proud 2016; Ma 2023). Indeed, AG compound was originally developed as a potential anti‐cancer therapeutic agent (Chen et al. 2011). Notably, substantial evidence has demonstrated that eEF2K activity is not required for cell survival and development under physiological conditions. For example, the eEF2K KO mice, in which the gene encoding eEF2 is deleted in the whole body, appear normal during development with a normal lifespan, and show normal performance in measurement of memory and synaptic plasticity (Gosrani et al. 2020; Kasica, Zhou, Jester, et al. 2022; Ryazanov 2002; Zimmermann et al. 2018). Consistently, no adverse effects on cognitive and synaptic function were observed in WT mice treated with eEF2K inhibitors (Kasica, Zhou, Yang, et al. 2022; Wang et al. 2024). Taken together, inhibition of eEF2K could be a “safe” therapeutic strategy without severe side effects, which is particularly important for patients with chronic diseases that usually require taking medicine for a long time. Furthermore, as mentioned in the introduction, eEF2K belongs to the unique *α*‐kinase family which does not share similar catalytic domains as conventional protein kinases (Drennan and Ryazanov 2004). Thus, eEF2K inhibitors, if properly developed, should not impact activities of those CPKs that are important for a broad spectrum of biological functions. The AG compound was identified from a chemical library using High‐Throughput Screening (HTS) and exhibits remarkable selectivity and potency against eEF2K in cell assays (Chen et al. 2011). AG is one of the most used pharmacological tools for the study of eEF2K‐related signaling regulation under physiological and pathological conditions. Recent studies in animal models demonstrated that synaptic failure and cognitive impairments associated with AD and DS can be alleviated by treatment with AG, presumably through the mechanisms of eEF2K inhibition (Kasica, Zhou, Yang, et al. 2022; Wang et al. 2024). However, the current studies indicate that AG has dose‐dependent effects on non‐eEF2K targets. Using the transgenic mouse model with global eEF2K knockout, we found that AG at a higher dose (10 μM) can still induce chemical LTP in the absence of eEF2K (Figure 1). Conversely, taking advantage of a novel transgenic mouse model with eEF2K overexpression (eEF2K cKI) (Jester et al. 2025), we demonstrated that overactive eEF2K was unable to blunt the chemical LTP induced by AG treatment (at doses of 5 and 10 μM) (Figure 2). Notably, overall de novo *protein* synthesis is significantly reduced in the hippocampus of the eEF2K cKI mice (Jester et al. 2025), which may contribute to the regulation of synaptic potentiation with AG treatment (Figure 2). Hippocampal LTP is one of the most intensively studied forms of synaptic plasticity and is widely considered as the cellular model for learning and memory (Bliss and Collingridge 1993). While declined LTP is well‐known to be associated with memory impairments, enhanced LTP is also linked to impaired cognitive function (Banko et al. 2005; Hoeffer et al. 2008). Moreover, eEF2K KO mice display normal synaptic and cognitive function (Beckelman et al. 2019; Kasica, Zhou, Jester, et al. 2022), providing further evidence to support the notion that AG compound alters synaptic function through eEF2K‐independent mechanisms, which should be taken into consideration for interpretation from studies using the eEF2K inhibitors.

What could be the eEF2K‐independent mechanisms associated with the AG‐induced chemical LTP? Previous studies in rats using pharmacological approaches demonstrated that field excitatory postsynaptic potential (fEPSP) potentiation induced by AG is dependent on the activity of p38 MAPK but independent of the activities of PI3K‐AKT, protein kinase C (PKC), protein kinase A (PKA), or MEK/ERK (Weng et al. 2016). Interestingly, they reported that AG‐induced LTP still persists in the presence of two structurally distinct protein synthesis inhibitors (Anisomycin and cycloheximide), suggesting a protein synthesis‐independent mechanism (Weng et al. 2016). Future in‐depth studies are warranted to elucidate the molecular mechanisms underlying the effects of AG compound on neuronal function. Meanwhile, our findings, together with other studies in rodent models using AG, suggest a new protocol for the induction of chemical LTP.

## 3 Detailed Methods

All protocols involving animals were approved by the Institutional Animal Care and Use Committee of Wake Forest University School of Medicine. Mice were kept in compliance with the NIH *Guide for the Care and Use of Laboratory Animals*.

Mice were housed in a barrier facility dedicated to transgenic mice at Wake Forest School of Medicine. The facility operates in accordance with the standards and policies of the US Department of Agriculture's Animal Welfare Information Center (AWIC), and the NIH Guide for Care and Use of Laboratory Animals. The facility is kept on a 12 h light/dark cycle, with a regular feeding and cage‐cleaning schedule. Both female and male mice were used at the age of 3–5 months. The eEF2K knockout mice (eEF2K−/−) were developed as previously described (Beckelman et al. 2019). The eEF2K knock‐in mice (eEF2K KI) were generated in collaboration with Dr. Dale Cowley, the director of the Animal Models Core Facility at the University of North Carolina at Chapel Hill. All genotyping was done by polymerase chain reaction (PCR).

Acute transverse hippocampal slices (400 μm thick) were prepared using a Leica VT1200S vibratome (Wetzlar, Germany) as previously described (Yang et al. 2016). The cutting solution contains (in mM): 87 NaCl, 25 NaHCO_3_, 2.5 KCl, 0.5 CaCl_2_, 7 MgCl_2_,1.25 NaH_2_PO_4_, 75 Sucrose and 25 Glucose. Slices were maintained for at least 2 h at room temperature prior to experimentation in artificial cerebrospinal fluid (ACSF) containing (in mM): 125 NaCl, 2.5 KCl, 2.0 CaCl_2_, 1.0 MgCl_2_, 1.25 NaH_2_PO_4_, 25 NaHCO_3_ and 25 glucose, bubbled with 95% O_2_/5% CO_2_. For electrophysiology experiment, hippocampal slices were transferred to recording chambers (preheated to 32°C) where they were superfused with oxygenated ACSF. Monophasic, constant‐current stimuli (100 μs) were delivered with a concentric bipolar microelectrode (FHC Inc., Bowdoin, ME) placed in the stratum radiatum of area CA3, and the field excitatory postsynaptic potentials (fEPSPs) were recorded in the stratum radiatum of area CA1. fEPSPs were acquired, and amplitudes and maximum initial slopes measured, using pClamp 10 (Axon Instruments, Foster City, CA). After 30 min of stable baseline recording, A484954 (Millipore, USA) was added to the perfusion solution. A484954 was made as a stock solution (10 mM) and diluted to the designated concentration (1, 5, and 10 μM) during the experiments.

Data are presented as a mean + SEM. Summary data are presented as group means with SE bars. For comparisons between 2 groups, a 2‐tailed independent Student's *t‐*test was performed using Prism 9 software (GraphPad Software). Two‐tailed paired *t‐*test were performed for within‐group analyses. For comparisons among more than 2 groups, 1‐way ANOVA was used with Tukey's post hoc tests for multiple comparisons. Grubb's test for outliers was performed on data, and any data points identified as outliers were excluded. Error probabilities of *p* < 0.05 were considered statistically significant unless otherwise noted.

## Funding

This work was supported by the National Institute on Aging (R01 AG073823, RF1 AG082388).

## Conflicts of Interest

The authors declare no conflicts of interest.

## Supporting information


**Figure S1:** Measurement of paired‐pulse facilitation (PPF) in hippocampal slices of eEF2K KO and eEF2K cKI mice treated with A484594 at 1 μM (A, B), 5 μM (C, D), and 10 μM (E, F). *n* = 2–12.
**Figure S2:** Treatment of acute hippocampal slices with A484594 decreases eEF2 phosphorylation. (A) Representative western blot images for p‐eEF2, total eEF2, and GAPDH at three different doses of A484594. (B) Significant decrease in immunoreactivity of p‐eEF2, normalized to total eEF2, with the 10 μM dose of A484594 compared to vehicle controls. (C) No change in total eEF2 levels, normalized to GAPDH, across doses of A484594. *n* = 3 for all groups. Error bars represent ± SEM. *p < 0.05, One‐way ANOVA with Tukey's post hoc.

## Data Availability

The data that support the findings of this study are available from the corresponding author upon reasonable request.
