# Supplementary material for: Synaptic Potentiation in Hippocampus by eEF2K Inhibitor A484954
Source: Hippocampus. 2026 Mar 27;36(3):e70091. doi: 10.1002/hipo.70091 (PMC13031159; doi:10.1002/hipo.70091)
Supplement: Supplementary file 1 — Figure S1: Measurement of paired‐pulse facilitation (PPF) in hippocampal slices of eEF2K KO and eEF2K cKI mice treated with A484594 at 1 μM (A, B), 5 μM (C, D), and 10 μM (E, F). n = 2–12. Figure S2: Treatment of acute hippocampal slices with A484594 decreases eEF2 phosphorylation. (A) Representative western blot images for p‐eEF2, total eEF2, and GAPDH at three different doses of A484594. (B) Significant decrease in immunoreactivity of p‐eEF2, normalized to total eEF2, with the 10 μM dose of A484594 compared to vehicle controls. (C) No change in total eEF2 levels, normalized to GAPDH, across doses of A484594. n = 3 for all groups. Error bars represent ± SEM. *p < 0.05, One‐way ANOVA with Tukey's post hoc. [file HIPO-36-0-s001.docx]

**

**

**Figure S1. Measurement of paired-pulse facilitation (PPF) in hippocampal slices of eEF2K KO and eEF2K cKI mice treated with A484594 at 1 µM (A-B), 5 µM (C-D), and 10 µM (E-F).** n=2-12.

**
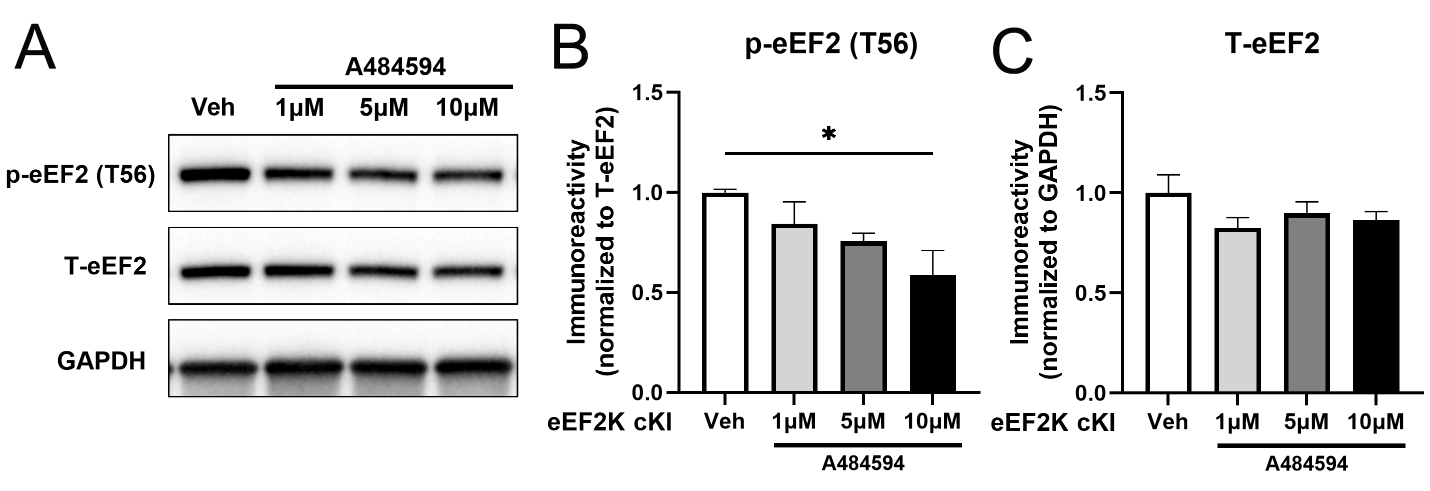
**

**Figure S2. Treatment of acute hippocampal slices with A484594 decreases eEF2 phosphorylation.** (**A**) Representative western blot images for p-eEF2, total eEF2, and GAPDH at 3 different doses of A484594. (**B**) Significant decrease in immunoreactivity of p-eEF2, normalized to total eEF2, with the 10μM dose of A484594 compared to vehicle controls. (**C**) No change in total eEF2 levels, normalized to GAPDH, across doses of A484594. n=3 for all groups. Error bars represent ± SEM. **p*<0.05, One-way ANOVA with Tukey’s post hoc.
